# CD24 activates the NLRP3 inflammasome through c-Src kinase activity in a model of the lining epithelium of inflamed periodontal tissues

**DOI:** 10.1002/iid3.40

**Published:** 2014-12-09

**Authors:** Wei Guo, Ping Ye, Hong Yu, Zhonghao Liu, Pishan Yang, Neil Hunter

**Affiliations:** 1 Department of Periodontology, School of Dentistry, Key Laboratory of Oral Biomedicine, Shandong UniversityShandong Province, China; 2 Institute of Dental Research, Westmead Millennium Institute and Westmead Centre for Oral HealthWestmead Hospital, Australia; 3 Microscopy Laboratory, Westmead Millennium InstituteWestmead Hospital, Australia; 4 Yantai Stomatological HospitalShandong Province, China; 5 Faculty of Dentistry, the University of SydneySydney, Australia

**Keywords:** CD24, inflammasome, NLRP3, periodontitis

## Abstract

Chronic periodontitis is characterized by perturbation of the epithelial attachment to the tooth with subsequent migration of the lining epithelium and formation of a cleft or pocket. This non-keratinized lining epithelium provides initial responses to bacterial products by signalling through receptors of innate immunity to activate inflammasome pathways. These comprise an intracellular network of regulatory and effector molecules leading to synthesis and activation of pro-inflammatory cytokines. Conversely, CD24 is characteristically strongly expressed by the pocket epithelium and is reported to function as an important negative regulator for danger signals, protecting tissues from excessive leukocyte activity. The objective of the study was to determine the impact of ligation of CD24 on expression of inflammasome components. An epithelial mimic of pocket epithelium was used to evaluate activation of the NLRP3 inflammasome pathway. Surprisingly, antibody ligation of CD24 enhanced expression of NLRP3 together with co-activators ASC and caspase-1 resulting in burst release of activated interleukin (IL)-18. Potent product inhibition was detected with IL-18 suppressing expression of NLRP3, ASC, and caspase-1. Scant distribution of these products within pocket epithelium compared with healthy gingival attachment provided indication of potential cycling of NLRP3 inflammasome expression. As subjects with mild chronic periodontitis have increased titres of serum antibodies auto-reactive with CD24 compared with those of subjects with severe periodontitis, a molecular mechanism for regulated expression of the NLRP3 inflammasome mediated by c-Src kinase activity, is proposed. This pathway could be regionally disrupted by products of pathogenic bacteria with profound downregulation in the dysbiosis associated with severe disease.

## Introduction

It is considered that gingival epithelial domains contribute to innate defences against pathogenic bacteria 1 and thereby regulate homeostasis of periodontal tissues in health and disease 2. Healthy gingival epithelium can be divided into three regions: oral epithelium (OE), sulcular epithelium (SE), and junctional epithelium (JE) 3. Non-keratinized sulcular and junctional epithelium are in close contact with bacteria in the gingival sulcus 4 and present the first line of defence 5,6.

Gingival epithelial cells sense and recognize pathogen- or danger-associated molecular patterns (PAMPs or DAMPs) 7 through stimulation of pathogen recognition receptors (PRRs) 1,4, with release of pro-inflammatory cytokines, chemokines, and defensins 8. Subsequent failure of the epithelial attachment to the tooth and migration of a markedly altered epithelium to form a cleft or pocket is characteristic of periodontitis 9, one of the most common chronic inflammatory conditions in humans 4,10. These changes are induced by the diffusion of bacterial products through the affected epithelium 11. Pathogenic bacteria, particularly *Porphyromonas gingivalis*, within the microbial biofilm release products that deregulate host immune responses 12. Marked banding of migration of protective polymorphonuclear leukocytes (PMNs) through the pocket epithelium suggests regional disruption of transmigratory capacity 13.

As extensive intracellular multi-protein complexes, inflammasomes play a central role in innate immunity. The complex is composed of NLR (Nod-like receptor) family proteins, products of 22 genes in humans 14,15. Three distinct subfamilies, the NODs (NOD1-5, CIITA), NLRPs (NLRP1-14, also called NALPs), and the IPAF subfamily, including IPAF (NLRC4) and NAIP 15 contribute to the inflammasome complex. Among inflammasomes, the pyrin domain-containing-3 (NLRP3) inflammasome is the most studied.

Gingival epithelial cells express a functional NLRP3 inflammasome 1 that can be activated by PAMPs 16. NLRP3 is assembled with the adaptor protein-ASC (apoptosis-associated speck-like protein) into a multi-protein complex that governs caspase-1 17 activation with subsequent maturation of pro-inflammatory cytokines interleukin (IL)-1β and IL-18 18–20 in the host response to infection and injury 21.

CD24 is selectively expressed at high levels by the epithelium associated with the healthy gingival attachment and pocket epithelium of periodontitis 22. Extensively glycosylated CD24 has recently been described as an important danger (or damage)-associated receptor, protecting tissues from excessive leukocyte activity 23. Bacterial sialidases downregulate CD24-mediated protection of tissues by presumptive cleavage of glyco-linkages, in a colitis model 24. CD24 critically mediates a protective effect against tissue injury 23 and facilitates expression and plasma membrane localization of tight junction components occludin, JAM-A, claudin-4, and claudin-15 that mediate increase of epithelial barrier function in gingival epithelial monolayers 25,26.

The objective of the present study was to firstly evaluate the distribution of inflammasome and tight junction components in the lining epithelium of both healthy and diseased gingival tissue sites. A model of the gingival epithelium was used to examine parameters for both induction and perturbation of inflammasome activation and tight junction assembly. These pathways were linked through a requirement for activation of c-Src kinase. Interleukin-18, as a secreted product of inflammasome activation, provided effective feedback inhibition to balance stimulation of the NLRP3 inflammasome pathway induced following ligation of CD24. Further, a periodontal pathogen *Porphyromonas gingivalis*, perturbed effective activation of the NLRP3 inflammasome and assembly of tight junctions, in the epithelial model. These findings provided the basis for the hypothesis that a key mechanism for pathogenic action in periodontitis is the disruption of inflammasome regulation and tight junction formation, in the lining epithelium.

## Materials and Methods

### Tissue samples

Gingival tissues were obtained with Ethics Committee approval and informed consent from 26 adult patients attending the periodontal clinic. Subjects had no record of periodontal therapy within the previous 3 years or antibiotic therapy within the preceding 6 months and were scheduled for extraction of teeth. Donors had no systemic diseases known to influence periodontal disease including diabetes. All patients had a detailed clinical record and radiographs. Tissues were classified by clinical and histological criteria. Clinically healthy gingival tissue was defined as minimally inflamed with a gingival index score of 0–2 and sulcus depth of 3 mm or less, and histological evidence of only a small number of chronic inflammatory cells in the sub-epithelial connective tissue. Chronic periodontitis was indicated by loss of 6–11 mm of attachment, formation of periodontal pockets with probing depths of 5–10 mm, resorption of alveolar bone, and the presence of numerous chronic inflammatory cells in the connective tissue. Prior to extraction two vertical incisions and a connecting apical incision 2 mm below and adjacent to the recorded probed site were made. Biopsies of the healthiest (minimally inflamed) and most diseased (periodontitis lesion) sites were obtained from each patient. Gingival tissues were snap-frozen in isopentane, cooled in liquid nitrogen, and 5 µm sections prepared for immunohistochemistry. Slides with sequential sections were stored in sealed boxes at −70 °C until required.

### Oral epithelial cell culture

The epithelial cell line (H413) derived from a human oral squamous cell carcinoma 27 displays stratified epithelial cell morphology and high CD24 expression in culture. H413 cell clonal lines were established using a limit dilution method as described previously 28. The cloned cells were cultured in Eagle's minimum essential medium (JMEM, Joklik modification, Sigma-Aldrich), penicillin/streptomycin (100 IU/ml, Sigma), and 10% fetal calf serum (FCS, CSL Limited, Victoria, Australia) at 37 °C in 5% CO_2_ 29. Cultures were harvested with triple express (replacement for trypsin, Invitrogen, Australia) in PBS and sub-cultured every 3 days.

### Immunostaining and confocal laser scanning microscopy

Frozen sections for immunostaining were fixed in 4% paraformaldehyde for 30 min at room temperature. After washing with phosphate-buffered saline (PBS), slides were placed in glycine-PBS for 10 min, washed in PBS and incubated in PBS containing 0.2% Tween 20 and 10% goat serum for 1 h at room temperature. After washing in PBS, sections were incubated with primary antibodies: rabbit anti-human occludin (5 µg/ml, Invitrogen), mouse anti-human NLRP3 (5 µg/ml, Abcam, UK), rabbit anti-human ASC, and caspase-1 (5 µg/ml, Abcam, UK) for 1 h at room temperature. As controls, isotype control mouse IgG1 and negative control rabbit IgG (Dakocytomation) were used as the primary antibodies. After washing in PBS, sections were incubated with 2 s antibodies, goat anti-rabbit IgG conjugated with Alexa Fluor 594 and goat anti-mouse IgG conjugated with Alexa Fluor 488 (Life Technologies, USA) for 1 h at room temperature. Sections were mounted in Prolong gold anti-fade reagent with DAPI.

Confluent H413 clone-1 cells (2 × 10^5^/cm^2^) in 8-well slide chambers (Corning) were treated with isotype control antibody or mouse monoclonal antibody (5 µg/ml) to CD24 peptide or anti-CD24 peptide plus c-Src inhibitor saracatinib (AZD0530, 1 µM, provided by AstraZeneca) or active recombinant IL-18 for 3 h, washed in PBS then fixed with 4% formaldehyde/PBS for 1 h, permeabilized with 0.1% Triton-X100/PBS for 15 min, and blocked with 10% goat serum/Tween-PBS for 1 h. The wells were incubated overnight at 4 °C with primary antibodies: rabbit polyclonal to NLRP3 and ASC (5 µg/ml, Abcam, UK), in 10% FCS/PBS at room temperature for 1 h in a humid chamber. After washing with PBS, fluorochrome-conjugated secondary antibody goat anti-rabbit IgG Alexa fluor 488 (Invitrogen), was added for 1 h at room temperature. For negative control, the primary antibody was replaced with control rabbit Ig (DAKO). Wells were washed with PBS and mounted onto glass slides using ProLong Gold antifade reagent with DAPI (Molecular Probes, Invitrogen).

Confocal images were obtained with an Olympus Fluoview (FV) 1000 equipped with Olympus FV 10-MCPSU (405 nm, 473 nm, 633 nm) and NTT electronic Optiλ (559 nm) lasers. Fields were selected at random [objective: Olympus 60X/1.35/0.15 (WD) Oil UPLSAPO] and the cells brought into focus under bright-field conditions. All fluorescence images prepared with confocal acquisition software (FV10-ASW 1.7) were stored and exported as TIF files.

### *Porphyromonas gingivalis* culture and challenge of oral epithelial cells

*P. gingivalis* strain (ATCC 33277) cultures were described previously 30. Briefly, culture maintained as frozen stock was inoculated into enriched CDC anaerobic broth, supplemented with haemin (5 μg/ml, Sigma) and menadione (5 μg/ml, Sigma) and grown in an anaerobic chamber (85% N_2_, 5% CO_2_, and 10% H_2_) for primary culture. Bacterial numbers were estimated by reference to the standard curve determined by absorbance at 600 nm of 1.0 (1 × 10^9^/ml) by spectrophotometry (Beckman, DU640) and collected in late exponential phase. Then *P. gingivalis* at a multiplicity of infection (MOI) of 100 31 to 1 epithelial cell, was added to confluent H413 cultures (5 × 10^6^ cells in T-25 cm^2^ flasks) and incubated with 10% fetal calf serum.

### RNA isolation and quantitative real-time RT-PCR for inflammasomes

Confluent H413 clone-1 cells were incubated with one of the following conditions for 3 h: 5 µg/ml of CD24 mouse monoclonal (ALB9) peptide antibody (IgG1, GeneTex Inc USA), which recognises a short non-glycosylated peptide sequence close to the site of GPI linkage to the protein core of the cluster-w4/CD24 antigen 22; treated with an IgG1 negative control antibody (5 µg/ml, DAKO, Denmark); treated with CD24 peptide antibody (5 µg/ml) plus c-Src inhibitor saracatinib (AZD0530, 1 µM); treated with recombinant IL-18 at 5 ng/ml in media; and treated with *P. gingivalis* strain (ATCC 33277) at a multiplicity of infection (MOI) of 100 31. Cells were harvested in 1 ml of Trizol reagent (Invitrogen) and RNA extracted as per the Trizol protocol. For reverse transcription, the First-Strand cDNAs were synthesized with oligo(dT)_12-18_ (Invitrogen), 10 mM dNTP (Promega), 5 × first strand buffer, RNaseOUT™ Recombinant RNase Inhibitor (Invitrogen) and SuperScript™ III Reverse Transcriptase (Invitrogen) according to the manufacturer's (Invitrogen) protocol.

Primers for genes encoding inflammasomes and tight junctions (Appendix Table 1) were designed using Oligo Explorer software (1.1.0) and synthesized by Integrated DNA Technologies (IDT, USA). Real-time RT-PCR analyses were performed by SYBR Green-based assays using the Stratagene MxPro-Mx3005P System. PCR reaction was conducted with 2 µl of diluted cDNA samples, 200 nM of each respective forward and reverse primer in 20 µl final reaction mixture with Platinum SYBR Green qPCR SuperMix-UDG (Invitrogen). cDNA samples isolated from non-manipulated H413 clone-1 cells were quantified by PicoGreen kit (Invitrogen) and used for constructing standard curves (2000–2 pg) by reference to the expression of the house keeping gene encoding β-actin. The PCR reaction for each gene was carried out in triplicate in 96-well plates, and initiated by activation at 95 °C for 2 min, followed by 40 PCR cycles of denaturation at 95 °C for 15 s, annealing and extension at 60 °C for 30 s. The results were analyzed using MxPro 4.10 software.

### Immunoassay-ELISA to quantify levels of IL-1β and IL-18

To measure extracellular and intracellular cytokine production of IL-1β and IL-18 from cells in the presence of anti-CD24 peptide antibody over a time course of 12 h, a standard sandwich enzyme-linked immuno-sorbent assay (ELISA) was introduced. Supernatants of test and control cultures or cells treated with 1% Triton in PBS for 10 min, were collected at 0, 1, 3, 5, 7, 9, 11 h, clarified by centrifugation and either analyzed immediately or aliquoted and stored at −20 °C. ELISA kits for cytokines were purchased from Abnova (Taiwan). According to the manufacturer's instruction, human IL-1β and IL-18 -specific monoclonal and polyclonal antibodies were pre-coated onto 96-well plates. The test samples were added to duplicate wells for 90 min at room temperature, and then biotinylated detection antibodies were added to the wells for 60 min at room temperature, and followed by washing 3x with PBS/0.05% Tween buffer. Avidin–biotin–peroxidase complex was added for 30 min at room temperature and unbound conjugates were washed away 5× with PBS/0.05% Tween buffer. HRP substrate TMB was used to visualize HRP enzyme reaction with a blue color product that changed to yellow after adding acidic stop solution. Absorbance at 450 nm was recorded within 30 min at room temperature after adding the stop solution. Standard samples were performed for each experiment. IL-1β and IL-18 concentration of the samples was calculated from the standard curve.

### Immunoblots for NLRP3 proteins

Proteins extracted in SDS sample buffer from H413 clone-1 cells treated with isotype control antibody or mouse monoclonal antibody to CD24 peptide (5 µg/ml) or CD24 peptide antibody (5 µg/ml) plus c-Src inhibitor saracatinib (AZD0530, 1 µM) or active recombinant IL-18 for 3 h, were separated by PAGE using gradient 5% to 12% mini-gels, transferred to nitrocellulose membranes (Bio-Rad) and blocked overnight with 3% bovine serum albumin (Sigma) in 0.1 M Tris buffered salts solution pH 7.4 (TBS). Blotted antigens were incubated with a mouse monoclonal anti-NLRP3 (1 µg/ml, Abcam), and a rabbit polyclonal anti-β-actin (0.1 µg/ml, GenTex) as a loading control in 0.05% Tween20/TBS for 2 h, washed and subsequently incubated with alkaline phosphatase (AP)-conjugated secondary antibody (goat-anti rabbit/mouse IgG, DAKO, Denmark) diluted 1:1500 in Tween20/TBS for 2 h. Bound antibody was visualized with AP substrate (Bio-Rad) after development of reactivity for proteins from control antibody, and anti-CD24 treated cultures under standardized conditions.

### Evaluation of tissue staining

**Figure 1:**
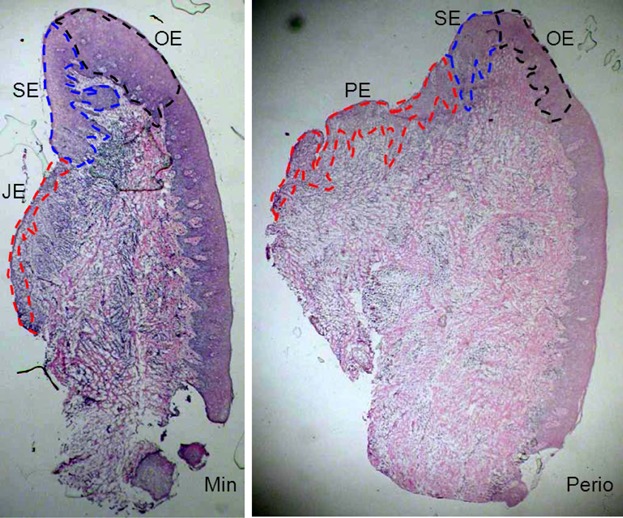
Gingival epithelial domains. The low magnification (×20) photomicrographs show the three different regions of the gingival tissues based on their architecture in minimally inflamed gingiva (Min) and periodontitis (Perio): JE (junctional epithelium)/PE (pocket epithelium), SE (sulcular epithelium), and OE (oral epithelium).

To ensure reproducibility, sequential sections from both sites of the same patient were processed in batches under uniform, standardized conditions. Gingival specimens were coded and examined as three regions (Fig. 1) for healthy group (minimally inflamed gingiva) and disease group (periodontitis) respectively, that is, oral epithelium (OE), sulcular epithelium (SE), junctional epithelium (JE), or pocket epithelium (PE).

The intensity of immunostaining was assessed on a visual analog scale ranging from 0 to 3, that is, 0 = − (negative), no staining or very little was detectable; 1 = +, weak staining; 2 = + +, moderate staining; 3 = + + +, strong staining. All sections were coded and scored by two independent observers who were calibrated to reference slides.

### Statistical analysis

All data were analyzed by paired *t*-test (mean ± s.d., two-tailed, 95% CI range) from at least three consecutive experiments for real-time RT-PCR, ELISA, and by unpaired *t*-test (mean ± s.d., two-tailed, 95% CI range) for tissue staining, where necessary. A level of *P *< 0.05 was accepted as statistically significant.

## Results

### Expression of NLRP3, ASC, caspase-1 and occludin in periodontal biopsies

Evaluation was based on sections from 18 patients using paired tissue biopsies that expressed good tissue orientation and intact epithelial domains (Fig. 1).

**Figure 2:**
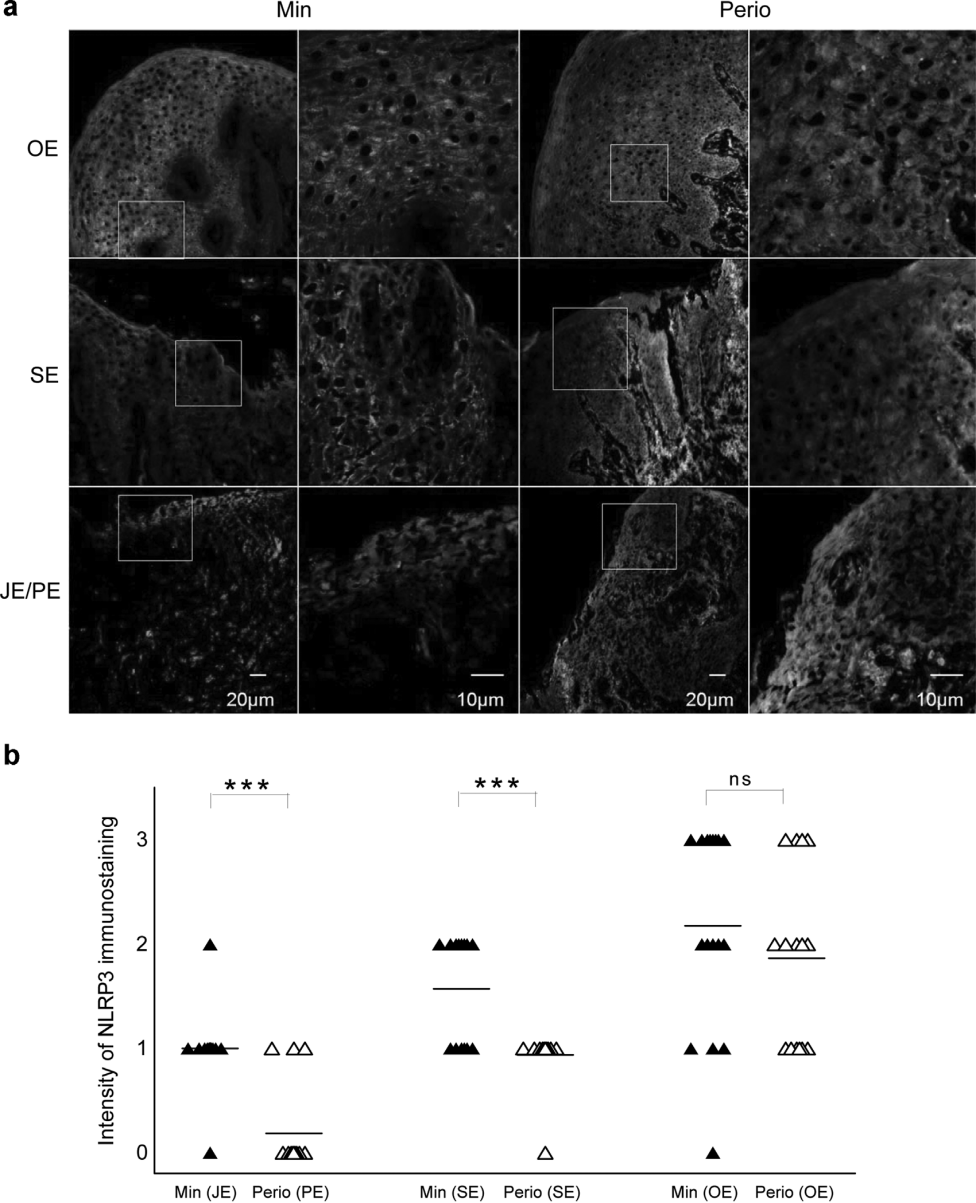
Immunostaining analysis for NLRP3. (a) Immunofluorescence staining of NLRP3 (green) in minimally inflamed gingiva (Min) and periodontitis (Perio). In the oral epithelium (OE), strong expression (+ + +) was observed in both Min and Perio. In the sulcular epithelium (SE), positive reaction (+ +) was detected near the surface and/or in the middle of the epithelium of Min, and weak immunoreactivity (+) was found near the base of the gingival sulcus of Perio. In the junctional epithelium (JE), weak staining (+) was apparent on the surface of the epithelium of Min, while no staining (−) was evident on the surface or in the internal structure of the pocket epithelium (PE) in Perio. (b) The scatter plots showing immunofluorescence staining of NLRP3 in minimally inflamed gingiva (Min) and periodontitis (Perio). Horizontal bars represent the median of the intensity of immunostaining (*** *P *< 0.001; ns, not significant).

Immunostaining was used to evaluate NLRP3 inflammasome expression. Strong expression of NLPR3 (Fig. 2a) was observed in external oral epithelia (OE) in both minimally inflamed (Min) and periodontitis (Perio) samples. The majority of immune reactivity was detected in the cytoplasm of the upper spinous layers of the gingival epithelium. Moderate expression of NLRP3 was detected in sulcular epithelium (SE) and junctional epithelium (JE) of minimally inflamed gingiva (Min), and sulcular epithelium (SE) of periodontitis (Perio) samples. Weak staining of NLRP3 was observed in the pocket epithelium (PE, Perio). Comparison of minimally inflamed and periodontitis groups indicated statistically significant differences in SE (Min/Perio) and JE/PE (Min/Perio) (*P* < 0.001, Fig. 2b). There was no significant difference in the oral epithelia (OE) between the two groups (Fig. 2b). Similar patterns to NLRP3 were observed for ASC and caspase-1 (data not shown).

**Figure 3:**
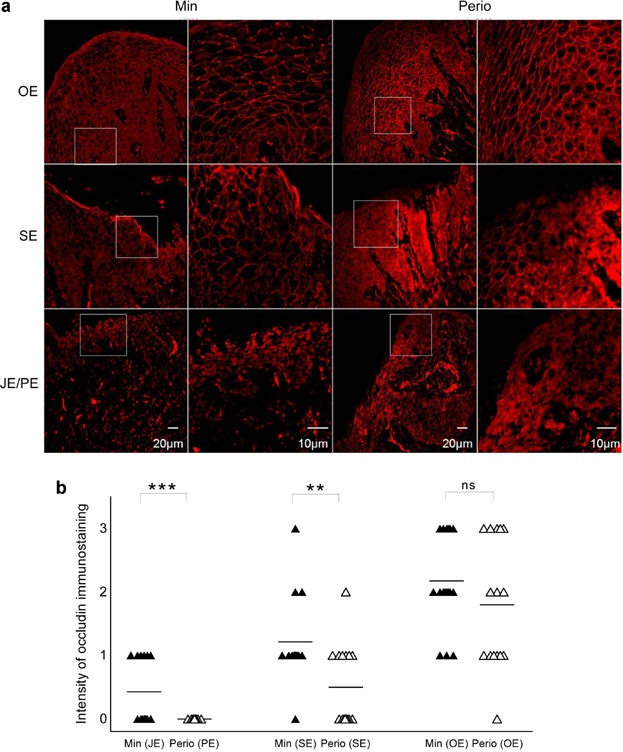
Immunostaining analysis for occludin. (a) Strong expression (+ + +) was detected in oral epithelia (OE). Moderate reaction (+ +) was observed in sulcular epithelium (SE) of Min, and faint staining (+) in gingival sulcus (SE) of Perio. A weak reaction (+) was evident in the junctional epithelium (JE) of Min, and no staining (−) in the pocket epithelium (PE) of Perio. (b) Again, a similar intensity pattern as for NLRP3. Scatter plots of occludin in minimally inflamed gingiva (Min) and periodontitis (Perio) (** *P *< 0.01, *** *P *< 0.001; ns, not significant).

Strong reactivity for occludin (Fig. 3a) was observed in external oral epithelia (OE) in both minimally inflamed gingiva (Min) and periodontitis (Perio) samples. Occludin was distributed at intercellular contacts throughout the epithelial strata. Only weak staining was apparent in the junctional epithelium (JE) of Min and pocket epithelium (PE) of Perio samples. Comparison indicated statistically significant differences in SE (Min/Perio) and JE/PE (Min/Perio) (*P* < 0.001, Fig. 3b). There was no significant difference in oral epithelia (OE) between the two groups (Fig. 3b).

**Figure 4:**
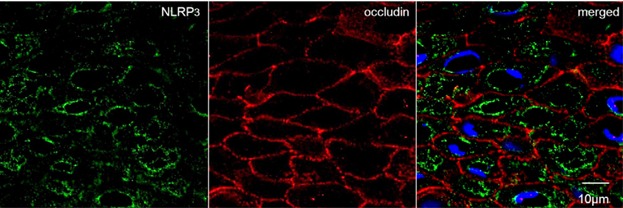
A co-localization image taken by confocal laser microscopy for NLRP3 (green) and occludin (red). NLRP3 protein located at cytoplasm in upper spinous layers, and occludin at cell contacts throughout the stratified epithelium.

Examination of patient tissues showed co-localization of NLRP3 and occludin by confocal laser microscopy (Fig. 4).

### Effect of CD24 peptide antibody, active recombinant IL-18 and *Porphyromonas gingivalis* infection on induction of genes encoding inflammasomes, pro-inflammatory cytokines, and tight junctions in oral epithelial cells

All quantitative real-time data were obtained as consistent results and performed with at least three different experiments with triplicate wells for each condition.

**Figure 5:**
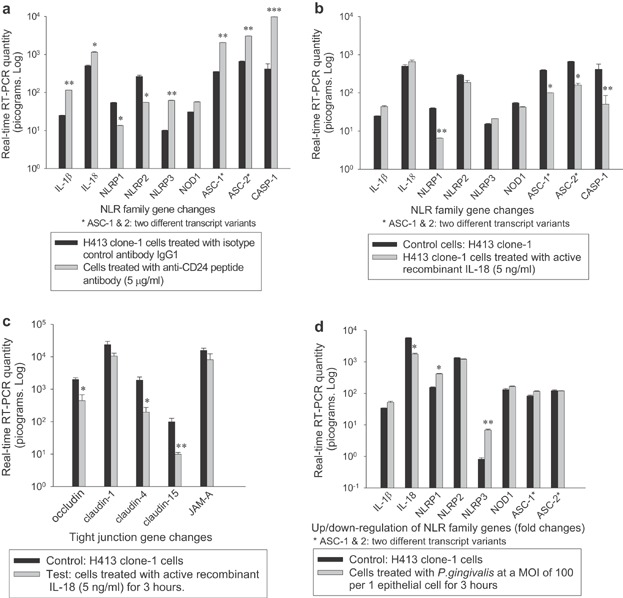
Quantitative real-time reverse transcription (RT)-PCR for gene expression of inflammasome and tight junction components in H413 epithelial cells in response to CD24 peptide antibody (a), active recombinant IL-18 (b and c), *P. gingivalis* (d) (* *P *< 0.05, ** *P *< 0.01, *** *P *< 0.001; paired *t*-test).

Significantly increased expression (Fig. 5a) of inflammasome genes encoding NLRP3 (6.16-fold), associated adaptor protein (ASC) (5.79- and 4.64-fold), caspase-1 (23.5-fold) and pro-inflammatory cytokines IL-1β (4.63-fold) and IL-18 (2.25-fold) was detected following incubation of H413 cells with anti-CD24 antibody. Expression for genes encoding NLRP1 and NLRP2 was reduced. There was no change in gene expression for NOD-1, only trace detection of gene expression for NOD-2 and no detectable expression of the gene encoding NLRC4 in this cell model (data not shown). Findings indicated that CD24 selectively activated the NLRP3 inflammasome and subsequently upregulated genes encoding ASC, caspase-1 and pro-inflammatory cytokines IL-1β and IL-18. Ligation of CD24 did not activate gene expression for NLRP-1, NLRP-2, NOD-1, or NOD-2 in H413 gingival epithelial cells.

Following exposure to active recombinant IL-18, there was potent inhibition of the suite of genes encoding the NLRP3 inflammasome (Fig. 5b). This suggested that released IL-18 inhibited NLRP3 inflammasome activity by negative feedback in H413 gingival epithelial cells. There was also significant inhibition of genes encoding tight junction components, occludin, claudin-4, and claudin-15 (Fig. 5c). This suggested a linkage between NLRP3 inflammasome and tight junctions.

Following challenge with *P. gingivalis* at a multiplicity of infection (MOI) of 100, expression of the genes encoding NLRP3 and NLRP1 was increased, but with the gene encoding ASC being discordant and the gene encoding pro-IL-18 was downregulated (Fig. 5d).

### CD24 peptide antibody elicits mature IL-18 cytokine production from oral epithelial cells

**Figure 6:**
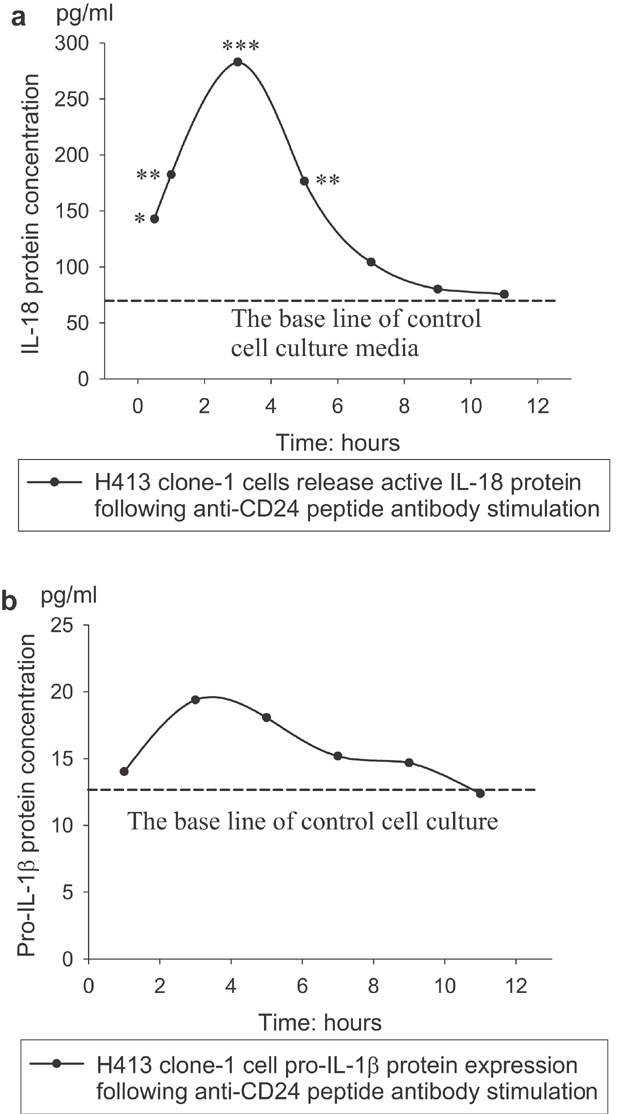
Immunoassay-ELISA to quantify mature cytokine IL-18 released from cell media (a) and synthesis of cytoplasmic pro-IL-1β from cell cytoplasm (b) over a time course, (*P < 0.05, **P < 0.01, ***P < 0.001; paired *t*-test).

Induction of release of active cytokines following ligation of CD24 was confirmed by ELISA. Data shown in Figure 6a demonstrate secretion of mature IL-18 from cultured cells stimulated by CD24 peptide antibody that was detectable by 30 min incubation (143 pg/ml), reaching a peak at 3 h (283 pg/ml) correlating with analysis of gene expression. Subsequently, levels declined to baseline.

There was no detectable mature (bioactive form) IL-1β released over the time course studied (data not shown). However, there was some increase in expression of the pro-form of the molecule (Fig. 6b) associated with increased gene expression (Fig. 5a) after cells were challenged with anti-CD24 peptide antibody.

### Inhibitor of Src kinases ablates the effect of anti-CD24 peptide antibody

**Figure 7:**
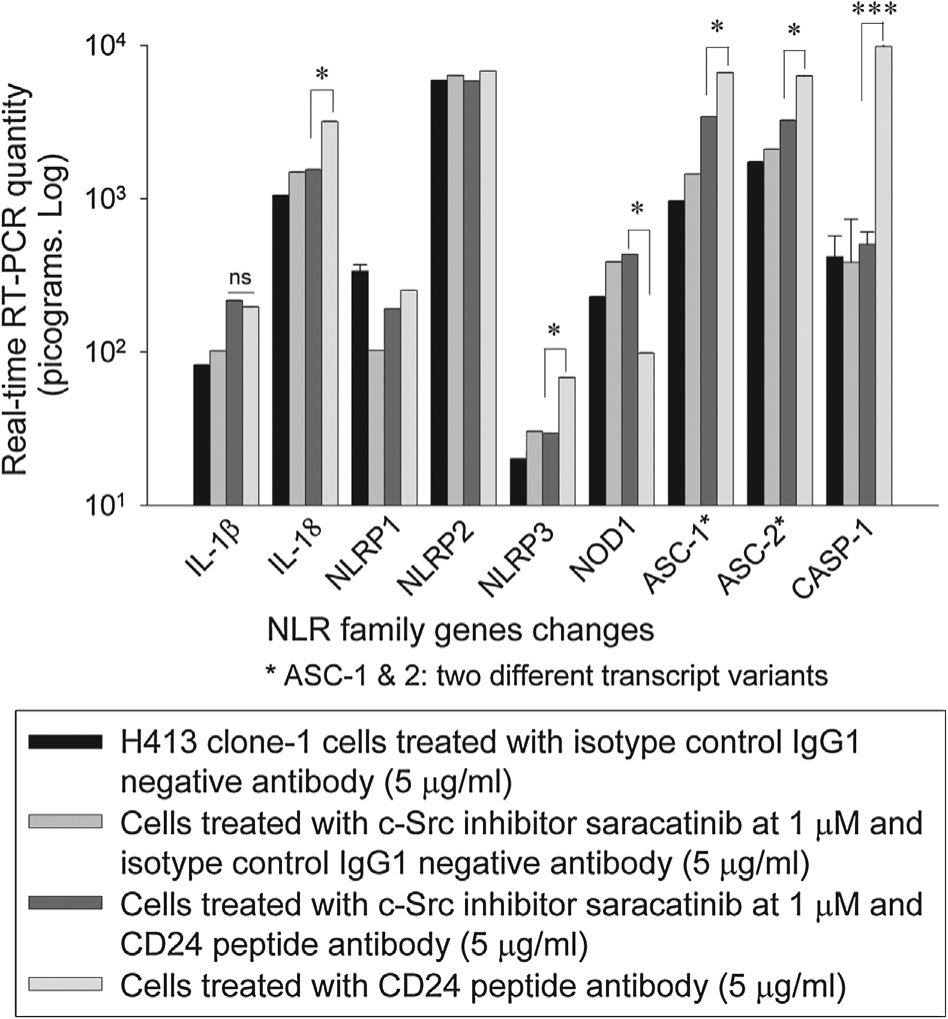
The bar graph shows significant downregulation of inflammasome genes encoding NLRP3, associated adaptor protein (ASC) and the pro-inflammatory cytokine IL-18 in H413 epithelial cells ligated with CD24 peptide antibody in response to Src kinase inhibitor for 3 h compared to H413 epithelial cells ligated with anti-CD24 peptide antibody only (* *P *< 0.05, ***P < 0.001; paired *t*-test).

The role of Src tyrosine kinase signalling pathway in the inflammasome response was assessed by addition of the selective tyrosine kinase inhibitor saracatinib (AZD0530). Cells were challenged with both anti-CD24 peptide antibody and Src kinase inhibitor saracatinib (AZD0530) for 3 h. There was significant downregulation of inflammasome genes encoding NLRP3 (2.32-fold), associated adaptor protein (ASC) (2.0-fold), caspase-1 (19.56-fold) and the pro-inflammatory cytokine IL-18 (2.07-fold), compared to cells challenged with anti-CD24 peptide antibody only (Fig. 7). This suggested that this pathway is involved in the process of CD24–NLRP3 interaction.

### The protein profile of NLRP3 in oral epithelial cells activated by anti-CD24 antibody

**Figure 8:**
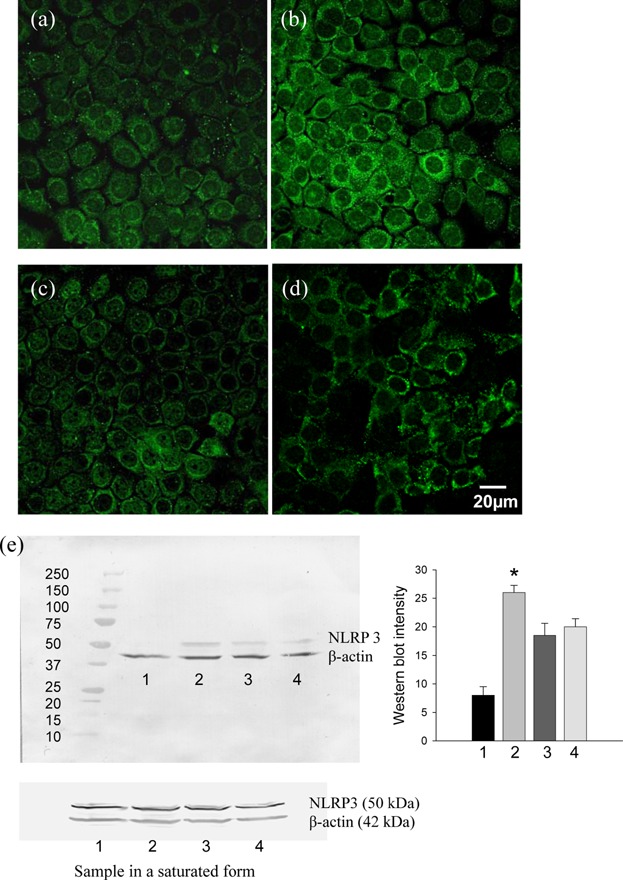
NLRP3 staining (a, b, c, and d) and Western blot (e). (a) and (e) lane-1: H413-1 cells treated with isotype control IgG1 negative antibody (5 µg/ml) for 3 h. (b) and (e) lane-2: cells treated with CD24 peptide antibody (5 µg/ml) for 3 h. An increased NLRP3 signal was observed by both confocal microscopy and Western blot (*P < 0.01) compared to control group. (c) and (e) lane-3: cells treated with CD24 peptide antibody (5 µg/ml) plus c-Src inhibitor saracatinib (1 µM). (d) and (e) lane-4: cells treated with active recombinant protein IL-18 (5 ng/ml) for 3 h. Reduced expression of NLRP3 was observed compared with cells treated with anti-CD24 peptide only.

To confirm upregulation of NLRP3 protein level in challenged oral epithelial cells, immunostaining and Western blots were performed. NLRP3 protein expression was increased (*P* < 0.01) (Fig. 8) corresponding with the response of affected periodontal tissues. Inhibition of c-Src kinase prevented this response.

When H413 cells were exposed to active recombinant IL-18 protein, a consistent inhibition of NLRP3 protein was observed in both immunostaining and Western blot compared to cells ligated with anti-CD24.

## Discussion

The H413 oral epithelial model used in the study was previously confirmed as having features comparable with those of periodontal epithelia 30. Importantly, both H413 clone-1 cells and junctional as well as pocket epithelia show characteristic high intrinsic expression of CD24 32. Ligation of CD24 with antibody selective for the peptide core of the molecule activates expression of the NLRP3 inflammasome including ASC and caspase-1, to facilitate secretion of mature IL-18. It was previously established that ligation of CD24 promotes barrier function of epithelial monolayers by upregulating expression of multiple tight junction components and localising these to form functional tight junction complexes 25 through a mechanism dependent on c-Src kinase activation 26. Src family tyrosine kinases are also important in the activation of innate immune responses through TLR, RLR, and NLR signalling pathways 33–35.

Control of c-Src kinase activity by saracatinib (AZD0530), a highly selective Src/Abl kinase inhibitor 36, prevented upregulation of NLRP3 inflammasome components that included dampening of release of active IL-18 in this cell model. Accordingly, findings indicated a link between activation of the NLRP3 inflammasome and tight junction formation mediated by CD24 in the epithelial model 33.

Focal distribution of NLRP3 was evident in external oral gingival epithelium (OE) for biopsies of healthy sites. This extended but with reduced intensity to sulcular and junctional epithelium (SE and JE) in the healthy group. In biopsies of disease sites the OE showed reactivity equivalent to that of OE in healthy sites whereas the pocket epithelium (PE) displayed weak or negative immunofluorescence staining for NLRP3 corresponding to control epithelial cells (no ligation with anti-CD24 peptide antibody). This finding is compatible with reports that subgingival microbiota downregulate gene expression for NLRP3 and IL-1β 37,38.

Occludin was shown to be distributed in a manner similar to NLRP3 but with varying cellular location from epithelial cell borders in healthy tissues to intracellular staining in PE. Occludin serves as a marker for tight junctions, playing a key role in assembly of the intercellular tight junction complex 39 and regulating migration of γδ intraepithelial lymphocytes 40. Occludin also regulates the release of apoptotic cells from epithelial monolayers 41 thereby preventing loss of barrier integrity. Effective tight junctions restrict passage through the epithelium, allowing only transfer of low molecular weight solutes 42. Accordingly, tight junctions form a key barrier to biologically active microbial products. Failure to contain microbial plaque biomass and penetration of microbial products are considered as key determinants of disease progression.

In the mucosa of the intestine, depending on context, activation of the NLRP3 inflammasome has been implicated in both protective effects and immuno-pathology 20. As key products of NLRP3 inflammasome activation IL-18 and IL-1β are produced as inactive cytoplasmic precursors, which are cleaved by caspase-1 to mature active forms 21. Unlike IL-1β, IL-18 precursor is constitutively present in blood monocytes and most epithelial cells 43,44. As a member of the IL-1 cytokine family 45 the main function of IL-18 during infection is to promote the secretion of other pro-inflammatory cytokines 46, to activate MHCII expression and to selectively downregulate gene expression for occludin with or without impairment of epithelial barrier integrity 47. Findings from the present study indicate that exogenous IL-18 has a variable action on the expression levels of genes encoding tight junction proteins. There was a trend for downregulation of the gene encoding occludin with more pronounced downregulation of the genes encoding claudin-4 and claudin-15 demonstrated to be essential components for effective tight junction formation in the epithelial cell model 30. Expression of genes encoding other claudin, zonula occludin and junctional adhesion molecule moieties was not discernably affected (data not shown).

**Figure 9:**
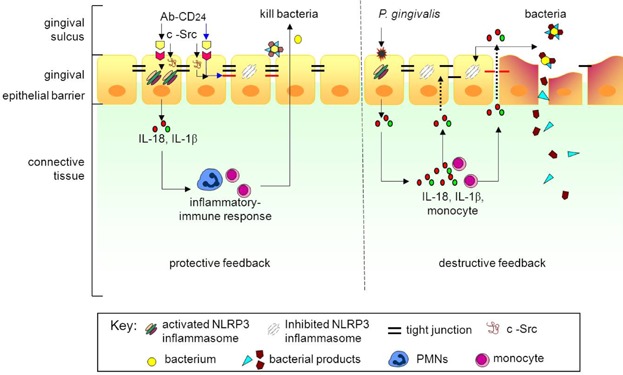
Illustration of a model for protective/destructive feedback through CD24-NLRP3 and *P. gingivalis*-NLRP3 interactions in healthy and diseased periodontal tissues. The non-keratinised lining epithelium (junctional epithelium) provides initial responses to bacterial products by signalling through receptors of innate immunity to activate NLRP3 inflammasome pathways (right panel). These comprise an intracellular network of regulatory and effector molecules leading to synthesis and activation of pro-inflammatory cytokines [IL-1β (green dots) & IL-18 (red dots)]. However, activated IL-18 potently suppresses NLRP3 and release of IL-18. Destructive feedback accelerates periodontal tissue damage. Conversely, CD24 is characteristically strongly expressed in the lining epithelium of inflamed tissues and functions as an important negative regulator for response to danger signals, protecting tissues from excessive leukocyte activity mediated by microbial activity (left panel).

It is reported that IL-18 levels in gingival crevicular fluid are elevated in sites of periodontal disease 48. This is compatible with downregulation of the NLRP3 inflammasome in affected epithelia as reported in the present study. In relation to the observations reported herein for an epithelial model where IL-18 potently suppresses NLRP3 and release of active IL-18, it is probable that IL-18 detected in crevicular fluid is derived from the inflammatory infiltrate. Interpretation of aspects of epithelial barrier response from the experimental model indicates that auto-reactive antibody ligation of CD24 would drive NLRP3 expression leading to tightly regulated levels of IL-18 within the epithelial environment and through a linked mechanism, an enhanced barrier through expression of functional tight junction complexes. It is probable that lectin-like receptors including from the commensal *Streptococcus gordonii* also stimulate through CD24 49 while sialidases from pathogens including *P. gingivalis* 50, downregulate this response. *P. gingivalis* also activates NLRP3 but also NLRP1 in the model system leading to a failure of co-response for ASC and downregulation of IL-18. That is, the organism perturbs inflammasome function. Coincident attack on epithelial intercellular junctional complexes by the proteases of *P. gingivalis* that downregulate barrier function 51, thereby facilitating an inflammatory response to microbial products, could also contribute to a flux of IL-18 release to further dampen both the NLRP3 inflammasome and effective tight junction formation. The proposed sequence illustrated in Figure 9 also provides an explanation in part for the observed lack of discernable tight junctions in the pocket epithelium**.**

## Appendix

**Table 1:** Primers used for real-time PCR

Genes	Oligos	Primers5′-3′	Expected amplicon size
NLRP3	F-primer	GCTGGACCTGAGTGACAAC	151 bp
	R-primer	GCTGAGTACCGAGGACAAAG	
NLRP1	F-primer	ACCATGGTAGTCCTGTTCAG	115bp
	R-primer	GCGAGTTTCCACTTAGGTC	
NLRP2	F-primer	AATGTCACTGCGTCTGAATC	154bp
	R-primer	CTAGGGAGGCACTGAGAAAG	
NOD-1	F-primer	TCTCAGACTCAGCGTAAACC	115bp
	R-primer	ATCGGTGATCTGGTTGTTG	
NOD-2	F-primer	TCTCTGTGCGGACTCTACTC	143bp
	R-primer	ATCCGTGAACCTGAACTTG	
NLRC4	F-primer	CAGGACTTGAATGGACAAAG	102bp
	R-primer	CAGAAAAGATGGGGTATGGT	
ASC-1	F-primer	AGGCCTGCACTTTATAGACC	174bp
	R-primer	GCTGGTGTGAAACTGAAGAG	
ASC-2	F-primer	GCTGCTGGATGCTCTGTAC	112bp
	R-primer	GGCTGGTGTGAAACTGAAG	
caspase-1	F-primer	GAAAAGCCATGGCCGACAAG	205bp
	R-primer	GCCCCTTTCGGAATAACGGA	
IL-1β	F-primer	GGCCCTAAACAGATGAAGTG	90bp
	R-primer	GTAGTGGTGGTCGGAGATTC	
IL-18	F-primer	GCATCAACTTTGTGGCAAT	161bp
	R-primer	CCGATTTCCTTGGTCAAT	
occludin	F-primer	GTCCAATATTTTGTGGGACAAGG	98bp
	R-primer	GGCACGTCCTGTGTGCCT	
claudin-1	F-primer	CAATGCCAGGTACGAATTTGG	135bp
	R-primer	TGGATAGGGCCTTGGTGTTG	
claudin-4	F-primer	TCATCGGCAGCAACATTGTC	116bp
	R-primer	GCAGTGCCAGCAGCGAGT	
claudin-15	F-primer	CCTTTGGCTTCTTCATGG	127bp
	R-primer	CAGAGGTTCTCGAAGATGG	
JAM-A	F-primer	CAAGTCGAGAGGAAACTGTTG	102bp
	R-primer	TCTGACTTCAGGTTCAGAAGAG	
β-actin	F-primer	ACTCTTCCAGCCTTCCTTC	216bp
	R-primer	GGAGCAATGATCTTGATCTTC	

F-primer: forward primer; R-primer: reverse primer.
